# Supplementary material for: Are carbon emissions trading and green financial instruments synergistic? -Comprehensive quantitative research based on content analysis
Source: PLoS One. 2024 Mar 7;19(3):e0298601. doi: 10.1371/journal.pone.0298601 (PMC10919601; doi:10.1371/journal.pone.0298601)
Supplement: S1 Data — (ZIP) [file pone.0298601.s001.zip › Data/地级市Z_F工作报告.zip!/║■▒▒/╧σ╤⌠╩╨/└┤╫╘╣1⁄2╓┌║┼í╛┬φ┐╦╩2╛▌═°í┐_2015.docx]

各位代表：

　　现在，我代表市政府向大会报告工作，请各位代表审议，并请政协委员和其他列席人员提出意见。

　　一、2014年工作回顾

　　过去的一年，面对结构调整“阵痛”加剧、实体经济艰难前行、困难挑战前所未有的复杂严峻形势，在市委的坚强领导下，全市上下主动适应经济发展新常态，攻坚克难抓改革，综合施策促增长，经济逆势而进，民生持续改善，社会和谐稳定，汉江流域中心城市建设展现出勃勃生机。

　　（一）经济增速全省领先，主要指标占比继续提高。预计全年地区生产总值增长9.8%，跨上3000亿元新台阶；规模以上工业增加值、固定资产投资、社会消费品零售总额、地方公共财政预算收入同比分别增长11.2%、22.5%、13.4%、30.1%，均高于全省平均水平，全省占比进一步提升。“双轮驱动”发展成效显著，高新区经济总量、创新发展能力分别跃居全国高新区23和29位，5个县（市、区）被评为“全省县域经济工作先进单位”。

　　（二）产业升级步伐加快，经济结构呈现积极变化。工业经济升级成效明显。传统产业技改投资、新兴产业项目投资占工业投资的比重同比分别提高6.4和2.1个百分点，新兴产业产值占规模以上工业产值的比重提高1.6个百分点，特色产业园区产业集中度提高2个百分点，新增1家产销过100亿元、2家过50亿元、10家过10亿元、50家过亿元的骨干企业和4家全省百强企业，成为全国产业集群区域品牌建设（汽车产业）试点地区、国家新能源汽车零部件创新集群示范基地，规模以上工业产值跃居全省第二位。服务业呈现跨越发展态势。云服务、电商、软件服务外包等新兴业态快速发展，成为国家二级物流园区布局城市、电子商务示范城市、信息惠民试点城市、云计算服务安全审查国家标准应用试点城市；文化旅游业融合发展，一批重大项目建成运营，“千古帝乡、智慧襄阳”城市品牌影响力日益提升。现代农业强市建设扎实推进。农产品加工业三年倍增计划深入实施，家庭农场、农民专业合作社、专业大户等新型农业经营主体快速发展，新增全国农民专业合作社示范社13个；农业综合生产能力进一步提升，连续三年获得“全国粮食生产先进市”称号，我市成为国家现代农业示范区，“中国有机谷”上升为省级战略。创新驱动发展战略深入实施。检验检测产业园初见雏形，2个国家级、2个省级检验检测平台即将投入运行，5家检测企业落户园区；新增19家省级研发机构、4家省博士后创新实践基地，新引进31个院士专家和大学生创新创业团队，新成立3个产业创新联盟，高新技术创业服务中心成为国家级中小企业公共服务示范平台；新增授权专利1747件，新登记科技成果258项，新认定高新技术企业56家，新增中国驰名商标和中国地理标志商标17件。

　　（三）重大项目加速落地，发展后劲明显增强。产业链招商强力推进，产业集聚效应明显增强。全市招商引资实际到位资金同比增长21.7%，新兴产业招商实际到位资金占比达32.6%，新注册10个投资过50亿元、67个过10亿元、109个过5亿元的重大项目，新引进6家世界500强企业，英菲尼迪全球核心配套商近地化配套企业集聚33家。项目建设强力推进，大项目大企业带动效应明显增强。全市实施亿元以上投资项目1103个，其中投资过10亿元的项目113个，亿元以上工业项目620个。一批新的经济增长点快速形成，英菲尼迪国产化、神龙公司扩能、骆驼集团襄阳公司二期等143个亿元以上工业项目竣工投产。一批事关襄阳长远发展的重大支撑性工程取得历史性突破，汉十高铁和郑万高铁即将开工并在东津并站，将把襄阳带入“高铁时代”；谷竹高速襄阳段建成通车，麻竹高速襄阳东段即将通车，实现了“县县通高速”；襄阳机场改扩建标志性工程“航站楼”、鄂北水资源配置工程开工建设。社会投资强力推进，民间投资拉动效应明显增强。全市新发展私营企业1.77万户，同比增长44.5%；民间投资占全市固定资产投资的比重达到81.45%，同比提高1.52个百分点。

　　（四）新型城镇化稳步推进，城乡一体化发展成效明显。中心城区功能和品位发生历史性变化。新区建设扎实推进，东津新区核心区基础设施实现全覆盖，起步区骨干路网基本建成，技师学院一期工程竣工，市民中心、医疗中心医技楼和门诊楼主体建成，华为华中云计算数据中心、华科工研院等产业项目快速推进；旧城更新有序实施，完成城市“两改”项目11个、改造27943户，综合整治“三无”小区325个；城市功能设施进一步完善，新建改建主次干道79条，襄樊大道综合改造完工，新开通公交线路12条150公里，新建城市供水、燃气、截污等市政管网132公里；生态体系建设强力推进，实施城市水系治理工程11项，新建改造公园游园11个、绿道40公里，新增城市绿地220万平方米，岘山成为国家级森林公园，山水园林城市特色进一步彰显；城市精细化管理水平明显提升，“五城同创”取得历史性突破，成功创建国家森林城市，通过了国家卫生城市检查验收。城乡一体化发展步伐加快。高起点编制了全域城镇化规划，宜城成为全国新型城镇化综合试点，“丹河谷”组群生态经济发展试验区、保康生态旅游试验区上升为省级战略；县（市）新城新区承载功能明显增强，产城融合深入推进；美丽乡村建设成效显著，“四化同步”示范镇建设取得积极进展，建成省级绿色示范村20个、宜居村庄30个，谷城堰河村、南漳峡口村成为全国美丽乡村建设试点，枣阳前湾村、南漳漫云村入选中国传统村落名录，尧治河村、石花镇等4个村镇入选“荆楚最美村镇”。生态文明建设力度明显加大。“绿满襄阳”行动、“碧水蓝天”工程、绿色低碳循环发展成效显著，中心城区空气质量优良天数比例达80%以上，汉江襄阳段水质保持二类水平，城市污泥及餐厨废弃物处理模式在全国推广并获“中国人居环境范例奖”，我市成为全国新能源示范城市和水生态文明城市建设试点，谷城县成为全国循环经济试点县，南河自然保护区成为国家级自然保护区。

　　（五）改革创新持续深化，市场活力加速释放。行政管理体制改革持续深化。简政放权深入推进，市级行政审批事项由145项减少到88项，在全省率先开展了工商登记便利化改革、建立了施工许可“一表制”，建成了具有国内领先水平的信息化审批平台，我市成为首批国家社会管理和公共服务综合标准化试点；高新区学习上海自贸区经验先行先试改革试点成效显著，在全省率先探索建立了“一枚印章管审批、一个部门管市场、一支队伍管执法”的“三个一”管理机制；在全省率先探索了“园办一体化”改革，完善了“五个一”的特色产业园区建设管理机制；重点领域综合执法体制探索有序展开，我市成为全国行政执法体制改革试点；统计基础工作进一步加强，圆满完成了第三次经济普查。财政管理体制改革成效显著。改变财政资金直接投入企业的方式，探索了建立产业发展基金支持产业企业的机制；初步建立了全口径财政预算制度，全面推行了部门预算和“三公”经费公开，深化了财政专项资金竞争性分配改革，实施了市区财政管理体制改革。金融改革创新取得新突破。在全省率先建立金融扶持实体经济机制，设立汉江产业基金，创新银政企合作机制，有力撬动了信贷投放，全市新增贷款额居全省地市州第一位；对接资本市场实现新突破，新三板和四板挂牌企业总数均居全省第一；互联网金融、融资租赁、直接债务融资、与资产管理公司战略合作等均取得积极成效，华中绿谷成为全国首家林业金融创新产业园。土地管理创新和农村产权制度改革稳步推进。农村土地承包经营权确权登记颁证试点工作基本完成，城乡建设用地增减挂钩试点工作得到国土资源部充分肯定，市县两级农村综合产权交易中心建成营运，全省首家区域性林权交易中心正式运行。公共服务体制机制创新扎实推进。医药卫生体制改革、乡镇公共文化管理体制改革走在全省前列，农村公共服务运行维护标准化建设试点成为全国示范。开放型经济体系建设加快。武汉新港襄阳陆地港、保税物流中心启动建设，襄阳至宁波港铁海联运班列实现常态化。

　　（六）社会建设全面加强，民生保障水平明显提升。民生投入力度持续加大。财政用于民生支出占总支出的比重达75.3%，同比提高1.7个百分点，“十件实事”、改善民生三年行动计划及农村“三万”活动年度任务全面完成。就业创业和社会保障工作力度持续加大。城镇新增就业11.3万人，创业带动就业5.4万人；城乡一体的养老、医疗保障机制取得实质性进展，城镇职工和城乡居民医保实现互转，保障性住房建设及住房公积金制度惠民力度进一步加大；扶贫、济困、助残、养老等工作都取得了积极成效，我市扶贫工作在全省考核中再次获得优秀等次，保康县脱贫奔小康试点工作在全省综合考评中连续五年名列第一，支援琼结、精河、鹤峰发展的各项工作取得了新成效。公共服务能力持续提升。教育质量和水平进一步提高，县（市）区全部通过省级县域义务教育发展基本均衡督导评估验收，高中教育水平继续领跑全省，“教育云”服务体系基本建立，高职院校办学水平和服务经济社会发展能力显著提高，湖北文理学院综合性大学创建取得积极进展；国家现代公共文化服务体系示范区创建扎实推进，汉江流域公共文化服务城市联盟被文化部评为创新示范品牌，我市成为国家现代公共文化服务标准化建设试点城市；卫生计生事业实现新发展，公共卫生工作走在全省前列，基层卫生服务体系进一步完善，医疗服务能力明显增强，出生政策符合率、出生人口性别比均超额完成省定目标；公共体育服务体系进一步完善，全民健身运动蓬勃开展。社会治理持续加强。标准化社区建设扎实推进，我市农村网格化服务管理、化解社会矛盾、“平安五小工程”建设经验在全国、全省推广，社会治安形势持续稳定，全市未发生较大以上生产安全和食品药品安全事故，我市成为全国安全发展示范试点城市、国家食品安全试点创建城市，再次获评全国综治优秀市。

　　一年来，我们紧紧围绕“转观念、转方式、转作风”加强自身建设，着力提升政府治理能力和水平。坚持以改革创新的举措破解发展难题，争创了一批全国全省改革示范试点；注重以法治思维和法治方式开展政府工作，自觉接受市人大、市政协和社会监督，认真办理人大代表议案、建议和政协建议案、提案，法治政府和法治城市建设全省领先；扎实开展党的群众路线教育实践活动，下大力气解决人民群众反映强烈的突出问题；严格落实政府系统党风廉政建设主体责任，坚决贯彻中央八项规定精神，深入推进“四风”问题治理，切实加强行政监察、审计监督和行政问责，有力促进了政风行风持续转变。

　　各位代表，过去的一年，我们面临的困难远超预期，挑战前所未有。全市广大干部群众同心协力、共克时艰，共同开启了汉江流域中心城市建设的新征程。在此，我代表市人民政府，向全市人民，向各位人大代表、政协委员，向各民主党派、工商联、人民团体和无党派人士，向驻襄人民解放军、武警官兵和公安干警，向所有关心支持襄阳发展的同志们、朋友们，表示衷心的感谢，致以崇高的敬意！

　　回顾过去一年的工作，我们清醒地看到，新常态下我市经济社会发展还面临许多困难和挑战。主要是：经济下行压力持续加大，实体经济困难加剧，保持持续较快发展势头面临新挑战；国家以定向调控倒逼结构调整的力度持续加大，我市传统产业比重过高，农业基础薄弱，战略性新兴产业、现代服务业支撑不足，推进产业转型升级面临新挑战；国家规范地方政府债务管理、推进预算制度改革、清理规范优惠政策等一系列措施出台实施，保障要素供给、推进招商引资和项目建设面临新挑战；财政收入增速放缓，刚性支出大幅增加，保持财政收支平衡面临新挑战；营商环境不优、创新创业活力不强、对外开放水平不高的问题还很突出，持续深化改革开放面临新挑战；覆盖城乡的社会保障和公共服务体系还不完善，满足人民群众日益增长的民生需求面临新挑战。面对这些矛盾和问题，我们必须以非常作为适应发展新常态，奋力推动经济社会发展“破茧成蝶”。

　　二、2015年工作安排

　　展望新的一年，既要充分估计经济新常态带来的新挑战，更要牢牢把握新常态蕴含的新机遇。结构调整的新常态带来了产业转型的新机遇。国家一系列推进产业升级的政策措施密集出台、定向发力，有利于推动我市具有比较优势的传统产业向中高端发展，有利于推进我市具有潜在优势的战略性新兴产业做大做强，有利于促进我市服务业新业态加快成长，有利于我市以整市创建国家现代农业示范区为抓手建设现代农业强市。区域发展的新格局带来了重塑“七省通衢”交通枢纽地位的新机遇。襄阳地处“一带一路”、长江经济带等国家战略辐射区，尤其是3条干线铁路、6条高速公路以及襄阳机场改扩建、现代航运体系等重大基础设施的陆续开工，将显著提升我市区域性综合交通枢纽地位，打开对接国家战略的大通道。国家以中西部为重点的新型城镇化布局为我市城镇化发展模式转型带来了新机遇。襄阳是中西结合部的重要节点城市，必将获得更多的政策和项目支持。国家改革创新试点示范带来了集聚要素的新机遇。近几年我市相继开展了103项国家试点示范，体制机制创新的效应逐步释放，扩大了襄阳的城市影响力，形成了吸引投资、集聚项目的强大磁场。与此同时，近年来我市有一批重大基础设施项目陆续开工，一批重大产业项目相继投产，一批新产业新业态快速集聚，一批新的经济增长点加速形成。今天的襄阳，多重机遇叠加，多种能量汇聚，迎来了转型跨越的历史“窗口”。牢牢抓住、切实用好这些有利条件和机遇，未来的襄阳就一定能够实现发展能级的新跃升、综合实力的新跨越。

　　今年政府工作的总体要求是：深入贯彻党的十八大和十八届三中、四中全会精神，全面落实省委十届五次全会和市委十二届九次、十次全会部署，主动适应经济发展新常态，认真落实“三维纲要”，坚持“竞进提质、又好又快”的基本要求，放手发展规模、努力提高质量，以强烈的文化意识指导城市建设与管理、统筹城乡一体化发展，以提高经济发展质量和效益为中心，把转方式调结构放在更加重要位置，狠抓改革攻坚，突出创新驱动，强化风险防控，加强民生保障，确保经济增速持续领跑全省和汉江流域，确保主要经济指标占全省和汉江流域的比重持续上升，确保城乡居民收入与经济同步增长，努力开创汉江流域中心城市建设新局面，为湖北“建成支点、走在前列”作出更大贡献。

　　今年全市经济增长预期目标为：地区生产总值增长11%，规模以上工业增加值增长12%，全社会固定资产投资增长22%，社会消费品零售总额增长14%，地方公共财政预算收入增长15%，外商直接投资增长15%，出口总额增长15%，城乡常住居民人均可支配收入增长11%，计划生育、节能减排、安全生产、居民消费价格指数等约束性指标全面完成省定目标。

　　围绕实现上述预期目标，重点做好以下七个方面的工作：

　　（一）着力扩大有效投资，加快培育新的经济增长点

　　紧扣提质增量推进项目建设。着力抓好省市重点项目建设，全力服务汉十高铁、郑万高铁及高铁站配套工程、蒙华铁路、枣潜等高速公路及国省干线、鄂北水资源配置工程等国家和省重大项目建设，加快推进襄阳机场改扩建、襄阳新港、雅口和新集航电枢纽、湖北能源路口电站、大唐热电联产、华润安能燃气机组等一批重大基础设施和重大能源项目，突出抓好英菲尼迪产业园、东风井关产业园、三环集团产业园、骆驼集团产业基地、东津新区云计算产业园等一批重大产业项目，确保30个以上省市重点项目竣工、180个亿元以上工业项目投产。对接国家重点投资领域、长江经济带发展规划纲要、国家“十三五”规划、国家产业投资基金、国家老工业基地改造政策、国家重大改革部署，积极策划争取一批国家重大投资项目和改革创新试点。

　　突出产业链培育强化招商引资。进一步创新招商引资方式，完善“六个一”招商机制，推动招商力量向产业链集聚、向特色产业园区集中，加快推进招商引资由单纯引企业向引产业及引资引智相结合转变、由单纯靠政策招商向营造产业配套环境招商转变、由全员招商向专业招商和以商招商转变、由全方位招商向定向定点精准招商转变。力争全年引进10个投资过50亿元、80个过10亿元、120个过5亿元的大项目，实际投资额突破1600亿元。

　　强化扶持措施推进大众创业。积极创建国家创业型城市，掀起大众创业的新浪潮，打造经济持续增长的新“发动机”。全面落实国家扶持小微企业、促进大众创业的政策措施，加快建设创业基地，着力提升国家级创业服务中心、国家级和省级创业孵化园、大学科技园、大学生创业园、海归创业园的载体功能，努力为每个有创业愿望的人提供自主创业的空间。重点支持大学生创业，引进高层次创业培训机构开展专业化创业辅导，依托汉江产业基金设立大学生创业基金，引导金融和创投机构扶持大学生创新创业。力争全年新发展小微企业7000家，新增大学生创业者1000名、带动就业5000人以上。

　　（二）着力优化产业结构，加快打造襄阳经济升级版

　　以新技术新产业推进制造业向中高端迈进。深入实施传统产业改造提升计划。牢牢把握工业4.0时代来临的新趋势，加大产业基金跟进、金融信贷支持力度，集中支持企业实施高端化、低碳化、数字化、智能化改造，重点抓好“百家企业技术改造示范工程”，力争技改投资占工业投资的比重达到40%以上。深入实施新兴产业倍增计划。大力支持纯电动和混合动力为主的新能源汽车、云计算为主的新一代信息技术、智能制造为主的高端装备制造等优势新兴产业做大做强，促进节能环保、生物医药、新能源新材料等特色新兴产业加速集聚，力争增加值占规模以上工业增加值的比重提高2个百分点。深入实施千亿产业培育计划。以推进英菲尼迪量产、乘用车新车型投放、专用车高端化规模化发展、整车零部件近地化配套为重点，进一步推动汽车及零部件产业延伸链条、壮大规模；以推动产品创新、管理创新、商业模式创新为重点，大力促进医药化工、纺织服装等产业转型升级；以国家城市矿产示范基地为依托，加快培育废旧金属、汽车、塑料、电子电器等再生资源回收利用及深加工产业。深入实施优势企业培育计划。大力推进百家龙头骨干企业成长工程、百家高成长性中小企业星火工程、百家科技型企业成长工程等“三百工程”，大力支持骨干企业“走出去”实施并购重组、加快做大做强，力争全年新增1家产销过100亿元、1家过50亿元、10家过10亿元、20家过亿元的骨干企业，新增50家高新技术企业。

　　以新业态培育推进服务业跨越发展。加快发展大科技服务业，重点培育检验检测、研发设计、科技中介等新兴业态，加快建设检验检测园区，以国家级检验认证机构为核心吸引一批行业龙头企业研发设计机构集聚发展，着力打造区域性检验检测中心。加快发展大数据产业，大力实施“互联网+”行动计划，促进制造业与云计算、物联网等融合发展、创新发展，加快推进以华为、IBM、软通动力、惠普、中国移动等为核心的“襄阳云谷”建设，探索利用数据资源建设面向中西部地区金融、电网等重点领域的灾备基地。加快发展大外包产业，着力打造中部地区汽车信息、金融保险服务外包基地，加快建设国内高端软件服务外包承接地和再发包中心，积极创建全国服务外包示范城市。加快发展大物流产业，着力培育电子商务、物流配送、连锁经营等现代流通方式，规划建设电商城，打造电子商务创业公共服务平台，大力推进电子商务推广应用和电商产业集聚发展，扎实抓好国家城市共同配送试点工作。加快发展大旅游产业，坚持以“一城两文化”引领文化旅游发展，推动文化、科技与旅游深度融合，突出培育生态观光、休闲度假、文化创意、运动健身等旅游业态，加强旅游标准化建设，着力提升“千古帝乡、智慧襄阳”的品牌影响力。加快发展大健康产业，积极顺应消费升级新趋势，大力培育健康、养老、体育等健康消费新热点。

　　以产业集聚区建设推进产业集聚集群集约发展。进一步完善“五个一”机制，大力推进产业集聚区建设。以特色产业园区为载体，引导制造业集中集聚发展；以中心城区商圈为平台，加快建设现代服务业集聚区，促进襄阳中豪国际商贸城、新合作襄阳食品商贸物流城、竹叶山农产品交易中心、新发地百应仓储物流园投入运营；加快建设文化旅游业集聚区，重点推进隆中文化旅游区、岘山文化旅游区、古城文化旅游区、鹿门山文化旅游区建设，启动鱼梁洲汉水文化旅游区、米芾国际文化创意产业园建设。

　　以创新驱动产业转型发展。以研发平台建设推动产业技术创新。依托高新区、经开区，按照“1（工业技术研究院）+N（产业技术研究中心）”的模式，加快推进华科工研院和校地企共建产业技术研究中心建设，创建一批国家级和省级企业技术研发机构。以成果转化平台建设吸引高层次人才创新创业。实施科技成果转化工程，加快建设国家技术转移中部中心襄阳分中心、院士专家创新创业园、中科院湖北产业技术育成中心（襄阳中心）、襄阳科技成果产业化促进中心、国家“海智计划”基地、襄阳科技城等科技成果转化平台，深入实施“隆中人才支持计划”，力争在引进院士专家和国内外高层次创新创业团队上取得新的突破。以创客平台建设促进万众创新。出台政策措施，构建创客服务体系，打造创客空间，吸引各类创新人才集聚襄阳创客空间开展创新创造；鼓励企业建立“众包”模式，吸引创客参与企业研发，带动万众创新。

　　（三）着力转变农业发展方式，加快建设现代农业强市

　　加快构建新型农业经营体系。大力发展新型农业经营主体，积极引进和培育现代农业公司，大力发展家庭农场、农民专业合作社、专业大户，积极创办区域性农民合作联社；积极创新土地流转和规模经营方式，探索土地经营权流转信托、托管合作等经营模式，实现金融资本、工商资本与土地有效对接，推动土地经营权向新型农业经营主体集中，提高农业规模化经营水平；创新农业科技推广服务体制机制，建立“综合性、跨区域、信息化、大统筹”的农业综合服务机构，鼓励社会力量兴办各类经营性服务组织。

　　加快构建现代农业产业体系。深入实施农产品加工业三年倍增计划，扎实推进省级农产品加工园区建设，推动农产品加工业转型升级，力争新增2家国家级农业产业化龙头企业；深入推进林业产业三年倍增计划，加快培育特色林产业；坚持“一村一品”、“一乡（县）一业”，着力推进农村一二三产业融合发展。

　　加快创建国家现代农业示范区。以创建全国绿色安全食品生产示范区为目标，大力推进“中国有机谷”建设，着力在推进核心区基础设施和有机特色农产品生产基地建设上取得重大进展；加快建设鄂北岗地现代粮食产业示范区，认真落实解决易旱地区三年行动计划，大力实施新型沃土、现代种业、现代农机推广应用、高产农田建设等工程，提高农业综合生产能力；加快建设汉江生态农业经济带，重点发展以生态种养、立体种养等模式为重点的循环农业；加快建设“襄（州）枣（阳）宜（城）”现代农业示范区核心区、樊城都市农业示范区，支持谷城全国休闲农业和乡村旅游试点县建设。探索发展智慧农业。

　　（四）着力提升城镇化质量，加快城乡一体化进程

　　坚持以新区建设和旧城更新提升城市承载能力。更加注重推进城市新区产城融合，集中力量建设东津新区“一核一带”，实现起步区和产业园区基础设施全覆盖，确保6000户被征迁居民全部入住安置房，市民中心、优质高中、医疗中心一期工程、华为华中云计算数据中心、华科工研院等项目投入营运；统筹推进庞公新区、樊西新区建设，加快推进县（市）新区建设。更加注重改善人居环境，加快推进城市棚户区改造，稳步推进专业市场外迁和工业企业转迁。更加注重提升城市功能，重点抓好路桥建设，大力推进城市供电、供水、燃气、排水、污水处理等市政工程，有序实施地下综合管廊工程，进一步完善城市路网、公共交通网和市政管网系统功能。更加注重提升城市品质，扎实推进文化元素融入工程、“活水入城”工程、生态绿地系统改造升级工程，新增城市绿地100万平方米，加快构建“500米见绿、1000米见园、2000米见水”的绿色生态格局，进一步彰显“一城两文化”特色。更加注重提升城市管理水平，完善大城管体制，推进城市管理精细、精密、全覆盖；推动智慧城市建设，启动智慧交通、智慧城管、智慧社区建设试点；进一步巩固和扩大“五城同创”成果。

　　坚持以规划引领和试点示范推进城镇化建设。创新城镇规划管理体制机制，出台实施市域新型城镇化规划，推进市县“多规合一”，完善空间规划体系，健全小城镇规划和管理职能。优化城镇化空间格局，科学划定城市边界，加快推进河谷组群、枣阳副中心、襄宜南一体化、保康生态旅游试验区发展。扎实开展新型城镇化试点，重点抓好宜城全国新型城镇化综合试点、襄城全域城乡一体化试点。以完善基本功能、覆盖基本服务、优化功能结构、强化产业支撑为重点，加快建设“四化同步”示范镇；扎实抓好整镇推进美丽乡村建设试点，大力开展村庄环境整治，实施城乡生活垃圾收集清运处理体系、农村公共服务平台建设等工程。以强烈的文化意识推进城镇化建设，着力在规划建设上强化文化引领、体现城镇文化特色，加强古城、历史文化街区和传统村落的保护利用，着力培育有历史记忆、文化脉络、地域风貌的特色城镇和村庄。

　　坚持以体制机制创新推动城镇化建设。全面实施居住证管理制度，积极探索农业转移人口市民化成本分担机制，建立要素支持与农业转移人口挂钩机制，加快推进农业转移人口市民化，逐步推进城镇基本公共服务覆盖城镇常住人口。探索建立多元化可持续的城镇化建设资金保障机制，建立规范的政府举债融资机制，推行政府与社会资本合作模式，多措并举拓宽城镇化投融资渠道。扎实抓好农村公共服务运行维护机制建设及标准化试点工作，进一步提高村级公共服务水平。

　　（五）着力推进改革开放，加快激发市场和社会活力

　　深入推进行政管理体制改革。持续深化简政放权，学习借鉴上海自贸区经验，深化高新区先行先试改革，在全市推广“三个一”的管理模式，加快构建社会信用体系；建设“政务云”平台，设立网上政务服务“超市”，推广应用以“四个大厅、两个平台”为主体的审批信息化系统，打造部门信息共享、协同联动的“线上政府”，扎实抓好国家社会管理和公共服务综合标准化试点工作，打造襄阳政务服务品牌。积极稳妥推进市县政府机构改革和事业单位分类改革，有序实施“区镇合一”体制改革。

　　着力推进财政和国有资产管理体制改革。深入推进预算制度改革，加快建立事权和支出责任相适应的市区财政管理体制，深化财政专项资金竞争性分配改革；深化公共服务提供方式改革，大力培育社会组织，扩大政府购买服务范围；强化政府债务管理，建立政府性债务管理机制、风险预警和处置机制。以管资本为主创新国有资产监管体制机制，以探索混合所有制和员工持股为重点深化国有企业改革，推进国有企业战略重组，着力解决国有企业历史遗留问题。

　　大力推进投融资体制改革。转变财政资金支持产业发展方式，组建汉江产业基金管理公司，与金融机构和专业基金公司合作设立若干子基金，变财政直接支持企业为基金跟投，撬动金融资本、吸引社会资本投入。鼓励金融机构创新金融产品和服务，引导信贷资金精准灌注实体经济，力争全年银行业金融机构贷款新增300亿元；建立科技金融服务中心，引导金融机构支持创新创造；推进农村金融创新，加快构建“合作社+金融”的新型农村金融服务体系。大力推进企业对接资本市场，力争全年有1家企业在主板上市、15家企业在新三板挂牌、20家企业在四板挂牌。大力发展股权投资，设立汉江天使投资基金、小微企业股权投资基金，积极探索私募股权众筹模式，吸引国内外知名股权投资机构集聚发展，着力打造区域性股权投资中心。积极培育新兴金融业态，大力发展互联网金融、融资租赁和直接债务融资，扩大与资产管理公司合作，着力突破保险融资和消费金融。规范发展民间金融，引导民间资本投向实体经济。推动建设农产品、苗木、城市矿产、光伏电站等要素市场交易平台。认真做好1%人口抽样调查工作，扎实推进投资统计制度改革。

　　积极推进土地及农村产权制度改革。大力推进城乡建设用地增减挂钩和城镇低效用地再开发利用试点。加快推进农村产权制度改革，全面推进农村土地承包经营权确权登记颁证，稳步推进农村集体建设用地使用权和宅基地使用权确权登记颁证，扎实抓好农村集体经营性建设用地入市和宅基地制度改革试点，逐步建立城乡统一的土地交易市场；完善农村产权流转交易平台，积极融入全省农村产权交易市场体系；深化农村集体产权制度改革，健全农村“三资”管理监督和收益分配制度。加快推进全国深化集体林权制度改革综合试验示范区建设，建立华中地区林权交易中心。创新集约节约用地机制，积极盘活存量用地，依法处置低效闲置用地。

　　加快构建开放型经济体系。积极对接“一带一路”，争取成为中铁集装箱公司汉新欧线路节点城市，大力拓展国际铁铁联运；启动建设襄阳国际陆港物流园区，加快建设“无水港”，积极推进武汉新港襄阳陆地港一期工程建设，力争开辟铁海联运新通道，打造区域性离港货物集散中心；积极申报建设保税物流中心，加快申报一类航空口岸，启动建设中国国家通报咨询中心襄阳研究评议基地和湖北国际旅行卫生保健中心襄阳分中心。加大引进外资力度，着力在引进境外世界500强企业上实现新突破。大力培育出口骨干企业，积极承接沿海加工贸易产业转移，提高加工贸易出口比重。大力支持优势企业抢抓“一带一路”建设机遇，加快“走出去”步伐。

　　（六）着力促进绿色发展，加快推进生态文明建设

　　深入实施“碧水蓝天”工程。扎实推进全国水生态文明试点城市建设，加强江河岸线环境综合整治，整体推进中心城区水系生态廊道建设，全面开展县（市）城区集中式饮用水源安全防护整治，完成6个污水处理厂、3个废水深度治理、12个规模化养殖场污染物总量减排等重点废水污染源治理工程。加强大气污染防治，实施改善空气质量整治工程，全面推行清洁生产，狠抓油气、机动车尾气、城市扬尘等污染治理，大力推进水泥生产线脱硝、燃煤发电机组脱硫脱硝除尘等改造项目，加强农作物秸秆综合利用和禁烧监管。

　　持续开展“绿满襄阳”行动。以构建“一城两带三网四区”的全域森林生态系统为重点，统筹推进城乡绿化一体化。深入推进高速公路、国省干道、县乡公路、乡村道路等绿色廊道建设，着力提升市域路网绿化层次；整镇推进乡村绿化；大力推进汉江流域、鄂北岗地、西南山地防护林建设，着力构筑生态安全屏障。全年完成植树造林15.2万亩，森林覆盖率提高1.5个百分点。

　　大力推进绿色低碳循环发展。积极创建国家可再生能源示范城市，大力发展节能建筑，实施大型公共建筑节能改造；大力发展低碳交通，积极推广应用新能源汽车；大力发展循环经济，重点建设再生资源利用、汽车零部件再制造、磷化工等循环经济产业链；加快推进餐厨废弃物无害化处理和资源化利用项目建设，全面建成收运系统和监管系统。

　　着力加强生态文明制度建设。编制生态市建设规划纲要，建立生态红线管控制度，加强对生态系统的保护和修复；认真落实最严格的耕地保护、水资源管理和水环境保护制度，细化汉江流域水环境综合治理考核办法；建立生态文明建设考核评价机制；建立领导干部生态文明建设责任制、问责制和终身追究制；严格执行环境保护法律法规，实行最严格的环境监管制度，坚决依法查处环境违法行为；完善环境风险管理制度，建立健全水环境污染、重金属污染、危化品污染等重大环境事件应急预案，确保无重大环境污染事故发生。

　　（七）着力保障和改善民生，切实加强社会建设

　　持续加大民生投入。全面完成改善民生三年行动计划和农村“三万”活动年度任务，扎实办好保障性住房、城区社区卫生服务中心、村卫生室、义务教育学校标准化、公共文化服务体系、养老服务体系、标准化社区建设，就业培训、城市公共交通改善、农村公路发展、“蓝天碧水”工程、农村饮水安全和城乡垃圾无害化处理、市区“三无”小区环境综合整治等13个方面的民生实事，着力织密民生安全网。

　　加快构建覆盖城乡的社会保障体系。更加注重保障基本民生。认真落实跨制度参保人员养老保险关系有序衔接和互转，建立被征地农民基本养老保险补偿机制，帮助残疾人普遍参加基本养老和基本医疗保险，实施全民参保登记计划，加快实现从“制度全覆盖”向“人口全覆盖”转变，进一步提高社会保险统筹层次和待遇水平；稳步推进新农合大病保险工作，将新农合人均筹资水平提高到450元左右；加快推进公共租赁住房、还建安置房建设和农村危房改造。更加关注低收入群众和特殊群体生活。推进城乡低保一体化建设，完善社会救助标准动态调整机制和临时救助制度，加大医疗救助力度，提高社会救助能力和水平；加强养老服务体系建设，提高五保对象供养能力，积极推行医养结合的社会化养老模式；加强农村留守儿童、妇女、老年人关爱服务体系建设，大力发展慈善、红十字会和残疾人事业。更加重视做好就业和扶贫开发工作。加强公共就业服务能力建设，加大就业培训力度，扎实开展全国新型职业农民培育整市推进试点，努力实现更高质量的就业；聚焦重点贫困人口，实施精准扶贫开发战略。

　　加快构建覆盖城乡的公共服务体系。推进各类教育科学发展。推动学前教育普及化、义务教育均衡化、高中教育优质化发展，加快建设普惠性幼儿园，扎实抓好农村寄宿制学校“五有”配套和义务教育标准化学校建设；推动职业教育改革，加快建设职教园区、职教联盟和实训基地，着力打造区域性现代职业教育中心；大力支持湖北文理学院创建综合性大学。加强公共文化服务体系建设。扎实推进国家现代公共文化服务体系示范区创建工作，加强市、县、乡、村（社区）四级公共文化网络标准化建设，全面实施“村村响”、“户户通”工程。加快发展卫生计生事业。深化医药卫生体制改革，扎实抓好医疗联合体建设和分级诊疗工作，积极推进县级公立医院改革，健全城区公共卫生服务体系，继续开展“四化”乡镇卫生院创建和社区卫生服务中心、村卫生室标准化建设；坚持计划生育基本国策，提高计划生育服务管理水平，强化出生人口性别比综合治理，促进人口均衡发展。加强全民健身公共服务体系建设，力争实现城乡基本健身设施全覆盖。

　　加快构建覆盖城乡的社会治理体系。加强城乡网格化管理，完善城市“一站四化”社区公共服务体系，推进农村网格化管理全面覆盖、规范运行。深入推进平安襄阳建设，着力创新立体化治安防控体系，进一步完善社会矛盾大调解和信访法治化体系，健全公共法律服务体系，加大法律援助力度，深入实施“平安五小工程”，加强流动人口和特殊人群服务管理，全面加强社会治安综合治理。强化食品药品安全监管，扎实开展国家食品安全试点城市创建工作，加快食品药品安全信息化平台和检验检测体系建设，推动食品药品安全社会共治，确保不发生重大食品药品安全事故。以创建国家安全发展示范城市为抓手，认真落实“党政同责、一岗双责、齐抓共管”制度，强化安全生产监管，严肃事故查处和责任追究，确保全市安全生产形势持续稳定好转。认真做好国防动员工作，大力支持国防后备力量建设，深化“双拥”共建。加强民族宗教工作，扎实做好援藏、援疆和对口援助鹤峰工作。

　　三、加强政府自身建设

　　新的一年，我们将按照市委关于全面推进法治襄阳建设的总体部署，深入推进依法行政，加快建设法治政府，自觉运用法治思维和法治方式深化改革、推动发展、化解矛盾、维护稳定。

　　（一）坚持依法履职，推进依法决策。全面落实权力清单制度，切实做到“法定职责必须为、法无授权不可为”，敢于担当、勇于负责，坚决纠正不作为、乱作为，坚决克服懒政、怠政。制定重大行政决策程序规则，健全公众参与、专家论证、风险评估、合法性审查、集体讨论决定的重大行政决策机制，推进新型智库建设，完善政府法律顾问制度，建立重大决策终身责任追究制及责任倒查制。扎实开展全省法治政府建设试点，坚持将深入推进依法行政、加快建设法治政府与改革发展稳定同部署、同落实、同考核。（二）改革执法体制，规范执法行为。加快推进执法重心和执法力量下移，在重点执法领域和经济开发区、经济发达镇建立综合执法体制。坚持严格规范公正文明执法，认真贯彻执行《行政诉讼法》，完善执法程序，建立执法全过程记录制，严格按照行政权力运行流程图开展行政执法，严格执行重大执法决定法制审核制度，完善涉企行政执法检查电子监察和重大行政处罚备案制度，健全行政裁量权基准制度，积极推行行政执法听证、公示和行政处罚信息公开制度，全面落实行政执法责任制，健全行政执法和刑事司法衔接制度，扎实抓好全省行政复议委员会试点工作。（三）自觉接受监督，强化权力制约。自觉接受市人大的法律监督、工作监督和市政协的民主监督，认真执行市人大的决议决定，主动听取市政协的意见建议，切实办理好人大代表议案、建议和政协建议案、提案，广泛听取各民主党派、工商联、无党派人士和人民团体的意见建议，主动接受社会公众和新闻舆论监督；加强对政府内部权力的制约，加强审计监督，防止权力滥用；全面推行政务公开，重点推进财政预算、公共资源配置、重大建设项目和社会公益事业建设等领域的信息公开。（四）创新工作方式，提高治理水平。以思维创新破解难题，主动适应经济发展的新常态。以互联网思维推动政府管理创新，树立政务服务“用户思维”，以公众需求为导向提供政务服务，以公众评价为标准改进政务服务；树立政府治理“社会化思维”，依托新媒体建立政府与公众互动机制，动员社会力量协同政府共同处理公共事务、参与政府治理。以市场化思维推动组织经济工作专业化，更加注重以科学的规划和政策引导产业发展，更加注重以市场机制推进经济工作，健全要素资源配置的市场化机制，促进优势资源向优势产业和企业集聚。（五）强化从严治政，建设清廉政府。巩固和扩大党的群众路线教育实践活动成果，持之以恒整治“四风”，以钉钉子的精神抓好作风问题整改，按照“三严三实”的要求加强政府自身建设。健全完善政府绩效考核机制，促进各级政府和部门多做打基础管长远的实事，确保关系发展全局的大事、关乎民生改善的要事干一件成一件。严格贯彻中央八项规定精神，深入贯彻厉行节约反对浪费条例，全面推行市县机关“三公”经费预算公开；严格财政供养人员编制管理，严肃查处“吃空饷”行为。落实从严治党、从严治吏要求，严格教育、管理、监督政府工作人员。创新反腐败体制机制，扎紧制度反腐的“笼子”，以“零容忍”的态度惩治腐败。建立政府机关权力监管清单，明确监管事项和监管责任，强化事后问责。保持坚强政治定力，加强纪律建设，切实履行“主体责任”，认真落实“一岗双责”，旗帜鲜明地支持纪检监察机关履行执纪监督责任，深入推进政府系统党风廉政建设，以优良的政风凝聚起改革发展的强大正能量。

　　各位代表！建设汉江流域中心城市，是全市人民的殷切期盼，是时代赋予我们的重大使命。让我们在市委的坚强领导下，凝聚跨越赶超的强大力量，迈开砥砺前行的坚实脚步，共同谱写建设汉江流域中心城市的时代华章！
